# Using a null hypothesis framework to test expectations of disparity in an adaptive radiation

**DOI:** 10.1098/rsos.250353

**Published:** 2025-10-29

**Authors:** Vhon Oliver Garcia, Cynthia Riginos, Simone Blomberg, Lyn G. Cook

**Affiliations:** ^1^ School of the Environment, The University of Queensland, St Lucia, Queensland, Australia; ^2^ Australian Institute of Marine Science, Townsville, Queensland, Australia

**Keywords:** adaptive radiation, null models, phylomorphospace, sea snake, adaptation

## Abstract

Adaptive radiations are expected to generate striking differences in species and morphological diversity between closely related groups. Not all hypotheses in evolutionary biology, including these observed disparities, are amenable to experimental manipulation or comparative phylogenetics. In some cases, the use of null hypotheses offers a way to test these scientific conjectures such that their premise can be rejected. We examine the *Hydrophis* sea snakes (Hydrophiinae: Hydrophiini), a putative example of adaptive radiation based on both its species and morphological diversity. We compared its observed species richness and morphological disparity with the closely related, yet species and morphologically depauperate clade, *Aipysurus-Emydocephalus*. We used phylogenetic null models and a phylomorphospace approach to test for significant differences in diversification rate and in morphological disparity between these two major sea snake lineages. We found no diversification rate differences between the *Hydrophis* and *Aipysurus-Emydocephalus* groups under an equal-rates Markov model of diversification. However, *Hydrophis* species occupy a significantly larger region of the morphospace than the *Aipysurus-Emydocephalus* group, consistent with previous conclusions that ecological specialization causes increased levels of morphological disparity. While the *Hydrophis* and *Aipysurus-Emydocephalus* lineages do not significantly differ in species numbers, the significant morphological disparity among *Hydrophis* species further upholds its adaptive radiation.

## 1. Introduction

Adaptive radiations are expected to result when a single common ancestor diversifies into many different species possessing ecological traits associated with available niches [1–4]. In putative adaptive radiations, discrepancies in species numbers between closely related groups are predicted as outcomes of increased speciation and/or reduced extinction rates of one group over the other. Additionally, the species formed are expected to evolve adaptations which correspond to distinct ecological niches, generating notable differences in morphological and ecological characteristics among members of the lineage [1,5–7]. Altogether, adaptive radiations can produce strikingly disparate patterns of species and morphological diversity between closely related groups.

A scientific hypothesis requires a way of being tested, such that its premise can be rejected. This is not an easy task in evolutionary biology where experiments are usually not possible. For example, it is not possible to run manipulative experiments to test hypotheses on whether some closely related groups indeed have significant differences in their levels of species and morphological diversity. One of the ways around this problem is to use null hypotheses to test the potential role of chance in generating specific patterns we observe [8]. This approach evaluates the significance of macroevolutionary observations by testing them against specific null hypotheses that accommodate the role of chance, divorced from any deterministic biological processes [9]. Random variation is an essential aspect of macro- and microevolutionary patterns and is a plausible and parsimonious explanation that should not be ignored. As such, any deterministic hypothesis that attempts to explain a pattern should use a test to determine if that pattern could be due to stochasticity [10].

**Figure 1.**
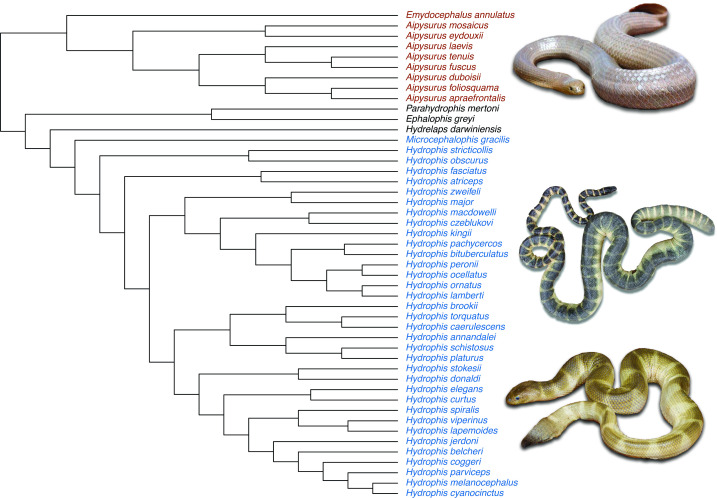
Ultrametric phylogenetic tree of true sea snakes (Elapidae: Hydrophiinae) showing the difference in species numbers between the two major sea snake clades (*Aipysurus-Emydocephalus*, red; *Hydrophis*, blue). Sea snakes showing *Aipysurus laevis* (top, photograph by Angus McNab) and morphological disparity in head size in *Hydrophis* species: *Hydrophis atriceps* (middle, photograph by Arne Rasmussen) and *Hydrophis curtus* (bottom, photograph by Stephen Zozaya) (Adapted and edited from Sherratt *et al.* [17]). Non-*Aipysurus-Emydocephalus* and non-*Hydrophis* taxa (black) were not included in the analyses as comparisons of disparity made by Lukoschek and Keogh [12] were between *Aipysurus-Emydocephalus* and *Hydrophis* clades.

In this study, we look at the putative adaptive radiation of the *Hydrophis* group of true sea snakes (Family Elapidae) [11–13]. True sea snakes (Hydrophiinae: Hydrophiini) are the most diverse extant group of marine reptiles, which diverged in the last ~6 million years [14]. These viviparous sea snakes form two major evolutionary lineages: *Aipysurus-Emydocephalus* and *Hydrophis* [12–14]. They descended from terrestrial Australo-Papuan elapids and represent a more recent transition into marine habitats, with the sea kraits (Hydrophiinae: *Laticauda*) being an earlier and independent transition [15]. Lukoschek and Keogh [12] published seminal work proposing that this group is an adaptive radiation based on (i) the topology of their phylogenetic hypothesis supporting a model of rapid speciation and (ii) *Hydrophis* sea snakes’ greater phenotypic diversity relative to the closely related *Aipysurus-Emydocephalus* lineage. Recent studies have concluded significantly elevated diversification rates in the *Hydrophis* group [16] and suggested that these sea snakes evolved a wide array of morphological modifications related to specific ecological roles [17] (figure 1). However, fixating on the speed of speciation conflates expectations from putative adaptive radiations with mere speciation [6]. Indeed, despite various methods used to identify temporal bursts of diversification, such bursts are not always detected in putative examples of adaptive radiation (reviewed in Glor [7]). Here, we test the two main claims of Lukoschek and Keogh [12] using a null-model framework to determine whether the *Hydrophis* group has (i) significantly higher number of species, and (ii) substantially greater degree of morphological disparity relative to the *Aipysurus-Emydocephalus* group than could have arisen by chance. We aim to test that the disparate distribution of phylogenetic and phenotypic diversity between the two major sea snake groups is unlikely to have been generated by chance alone. We evaluate the claims for the adaptive radiation of the *Hydrophis* clade based on the definition that adaptive radiation is the diversification of species with accompanying adaptations. Adaptive radiations are not necessarily rapid and cases where rapid speciation is observed do not necessarily qualify as adaptive radiations. This definition does not give precedence to explosive diversification as a criterion, which avoids conflation with mere speciation [1,6,7].

To test if the difference in species numbers between two clades of similar ages is due to differences in diversification rate, a null distribution of phylogenetic trees with a fixed number of taxa under an equal-rates Markov (ERM) process of diversification can be simulated [18]. The model is similar to a pure-birth model of diversification wherein speciation rate is held constant (*λ* = 1.0), with no extinction (*µ* = 0.0), and the ‘left’ and ‘right’ clades formed from the basal node of a simulated phylogeny have an equal rate of diversification, i.e. all possible ways to group a fixed number of taxa into two clades from the basal node are equally probable. We can then test our observed partition of taxa between two groups against a null distribution of phylogenetic trees and determine whether such differences could have arisen by chance under an equal rates model. Thus, in such tests, the null hypothesis is that there are equal rates of diversification present in both clades [8,19,20]. If it can be rejected, it means that the rates have not been equal.

Similarly, the apparent difference in morphological disparity among species within two closely related groups can be tested using their phylogeny projected into multivariate morphospace (i.e. phylomorphospace). Sidlauskas [21] demonstrated this approach to discriminate between two unequal morphological diversification scenarios: (i) unequal magnitude, where the more morphologically disparate group experienced greater morphological change per phylogenetic branch than the less disparate group; and (ii) unequal mode, where the more morphologically disparate group is inferred to have occupied a greater and more novel regions of the morphospace; thereby being more dispersed throughout the morphospace (i.e. lower lineage density) than the less morphologically disparate group which is then said to be more constrained (i.e. higher lineage density). Measures of magnitude and lineage density are expressed as ratios to relate the two groups under comparison. These ratios are then tested against a simulated null Brownian Motion model of morphological diversification to test if observed ratios of magnitude and lineage density are exceptionally unequal between diverse and depauperate groups compared with the null distribution. In tests for each scenario, rejecting a simulated Brownian Motion model of diversification infers that the compared groups evolved under different rates of morphological evolution (for scenario 1 in Sidlauskas [21]); and that at least one of the two groups has undergone a non-Brownian mode of morphological evolution such as adaptive radiation (for scenario 2 in Sidlauskas [21]).

## 2. Methods

### 2.1. Does the *Hydrophis* group have significantly higher number of species compared to *Aipysurus-Emydocephalus*?

We used a topology-based approach [18] and whole-tree methods [22] to test whether the difference in species numbers between the *Hydrophis* and *Aipysurus-Emydocephalus* clades may be associated with diversification rate variation or is indistinct from patterns generated under a phylogenetic null model. While there are many methods to estimate diversification rates, including those that attempt to infer speciation and extinction rates separately from phylogenies, these approaches can be misleading [23]. Here, we test the null hypothesis of equal diversification rate between the clades being compared.

First, we employed topological tests for diversification rate variation using clade size information for each sea snake clade obtained from published Squamate phylogenies [24,25], sea snake phylogenies [12,14,16,17] and a list of nominal species [26] (table 1). We then simulated 1 00 000 phylogenetic trees with a fixed number of tips under an ERM process of diversification (i.e. Yule/pure-birth model; speciation rate, *λ* = 1.0; extinction rate, *µ* = 0.0) using the sim.bd.taxa function in the TreeSim package version 2.4 [27] in R version 4.4.1 [28]. The total number of tips ( = taxa) for each simulation set was based on the total number of species counts across the two clades (*= N*; table 1). We then calculated the sizes (= species counts) of the ‘left’ and ‘right’ clades from the basal node for each simulated tree using the balance function in the R package ape version 5.8-1 [29]. We counted the number of simulated trees with ‘left’ clade sizes that are less than or equal to each of the lower numbers in each observed clade disparity and tested for significance at *α* = 0.05. For completeness, we also performed a similar test in which we simulated 1 00 000 trees using the speciation and extinction rates estimated for the snake Family Elapidae (*λ* = 0.146, *µ* = 0.046; [16]). The code used to perform these calculations from a set of simulated trees is available on GitHub (https://github.com/grcvhon/sea-snake-macroevolution).

**Table 1.** Topological tests of diversification rate variation do not support elevated diversification rate in *Hydrophis* relative to *Aipysurus-Emydocephalus* against an equal-rates Markov (ERM) null model and estimated speciation and extinction rates. *N* = total number of species in the source phylogeny.

source	*N*	species within each clade		*p*‐value	
		*Aipysurus-* *Emydocephalus*	*Hydrophis*	ERM (*λ* = 1.00; *μ* = 0.00)	Elapidae^a^ (*λ* = 0.146; *μ* = 0.046)
squamate phylogeny					
Pyron *et al*. [24]	27	6	21	0.46	0.61
Tonini *et al*. [25]	58	11	47	0.39	0.59
sea snake phylogeny					
Lukoschek and Keogh [12]	21	6	15	0.60	0.66
Sanders *et al*. [14]	35	8	27	0.47	0.61
Lee *et al*. [16]^b^	44	9	35	0.42	0.60
Sherratt *et al*. [17]	43	9	34	0.43	0.60
nominal species list					
Elfes *et al*. [26]	60	11	49	0.37	0.59

^a^ Speciation and extinction rates from Lee *et al*. [16].

^b^ Elapidae phylogeny

Second, we used the phylogeny of Elapidae in Lee *et al.* [16] to perform a single-tree analysis of diversification rate variation implemented in the software SymmeTREE version 1.1 [22]. SymmeTREE tests if a given tree has experienced significant shifts in diversification rates and, if so, localizes where these significant shifts have occurred. We estimated whole-tree diversification rate variation using two rate-shift statistics (*M*_Σ_ and *M*_Π;_ [18]) and a tree balance index (*B*_1_; [30]). Likelihood ratio tests were used to assess the location of significant rate shifts [22,31,32]. The probability of such a rate shift is estimated through the statistic, Δ_1_. A significant Δ_1_ value for a node infers that the more diverse clade subtending that node had a shift in diversification rate. Whole tree tests were performed using 1 000 000 simulated trees and under the taxon-size sensitive ERM (TSS-ERM) algorithm, which is most conservative with regards to the null hypothesis of no significant diversification rate variation [33].

### 2.2. Does the *Hydrophis* group have a substantially greater degree of morphological disparity compared to *Aipysurus-Emydocephalus*?

Following the methods in Sidlauskas [21], we used a phylomorphospace approach to test the null hypotheses that the *Hydrophis* and *Aipysurus-Emydocephalus* clades, despite having different levels of morphological disparity, (i) have experienced equal magnitudes of morphological change per phylogenetic branch and (ii) that their modes of morphological diversification are indistinct from a simulated null Brownian Motion model. To test each null hypothesis, we calculated the ratio of mean morphometric branch lengths (*M*-ratio) and the ratio of lineage densities (*D*-ratio) between clades.

To test the first hypothesis, we used the maximum clade credibility (MCC) tree and morphological trait dataset (body shape and body size) from Sherratt *et al.* [34]. These traits are described as in Sherratt *et al.* [34]: (i) body shape pertains to the relative girth of the snake (girth at 0.75 snout-vent length (SVL) divided by girth at neck); (ii) body size corresponds to the log-transformed maximum total length. We first ultrametricized the MCC tree and then extracted the clades of interest (i.e. *Hydrophis* and *Aipysurus-Emydocephalus*). For the *Hydrophis* clade, *Microcephalophis gracilis* was included since it is considered as *Hydrophis gracilis* in other sea snake phylogenies [25]. To calculate the observed mean morphometric branch length of each clade, we used a modified version of the phylomorphospace function in the phytools R package version 2.4-4 [35]. To obtain the observed *M*-ratio, the mean morphometric branch length for the *Hydrophis* clade (*M*_Hyd_) was divided by the mean morphometric branch length for the *Aipysurus-Emydocephalus* clade (*M*_AE_) (i.e. *M*-ratio = *M*_Hyd_/*M*_AE_). An *M*-ratio>1 means that the *Hydrophis* clade experienced a greater magnitude of morphological change than the *Aipysurus-Emydocephalus* clade [21].

For the second hypothesis, we calculated lineage density (*D*_1_; [21]), which takes the quotient of the sum of morphometric branch lengths and the volume of the morphospace hyperellipsoid. Since we only had two trait dimensions (= two morphospace axes), we calculated for the area of the ellipse using the ellipsoidhull function in the R package cluster version 2.1.6 [36], which returns a value for area when given two dimensions. We did not calculate lineage density using the alternative formula (*D*_2_) as it accounts for higher-order measures of the calculated volume (e.g. third-order volume or fourth-order hypervolume) [21]. To obtain the observed *D*-ratios, the lineage density of *Aipysurus-Emydocephalus* (*D*_AE_) was divided by the lineage density of *Hydrophis* (*D*_Hyd_) (i.e. *D*-ratio = *D*_AE_/*D*_Hyd_). A *D*-ratio>1 means that the *Hydrophis* clade occupies and uses a greater area of the morphospace (i.e. possessed greater efficiency in morphological innovation) than the *Aipysurus-Emydocephalus* group.

We assessed significance of calculated (i.e. observed) *M*- and *D*-ratios against a simulated null distribution generated under a Brownian Motion model of evolution. We used the 500 sample trees and morphological trait dataset from Sherratt *et al.* [34]. We first pruned taxa of non-interest (non-*Aipysurus-Emydocephalus* and non-*Hydrophis*) and ultrametricized all 500 trees.

We then randomly sampled 1 00 000 ultrametricized trees to generate our phylogenetic dataset. For our morphological trait dataset, values of each morphological trait were randomized independently across all taxa across both clades until we generated 1 00 000 sets of randomized observed trait values. Then, for each iteration (i.e. pair of ultrametric tree and set of randomized observed trait values), we fitted an intercept-only model using phylogenetic generalized least squares to estimate the root node value and used the variance to simulate new values for each morphological trait under a Brownian Motion model of evolution. We calculated for *M*- and *D*-ratios for all iterations (= 1 00 000) to generate a null distribution on which observed *M*- and *D*-ratios were tested for significance at *α* = 0.05. We calculated lineage densities (*D*_1_) for each clade and *D*-ratios using raw measurements of morphological traits as well as data transformed to a log_10_-scale. R scripts used to perform analyses in this section can be accessed at https://github.com/grcvhon/sea-snake-macroevolution.

## 3. Results

### 3.1. Testing the unusually high number of species in *Hydrophis*

**Figure 2.**
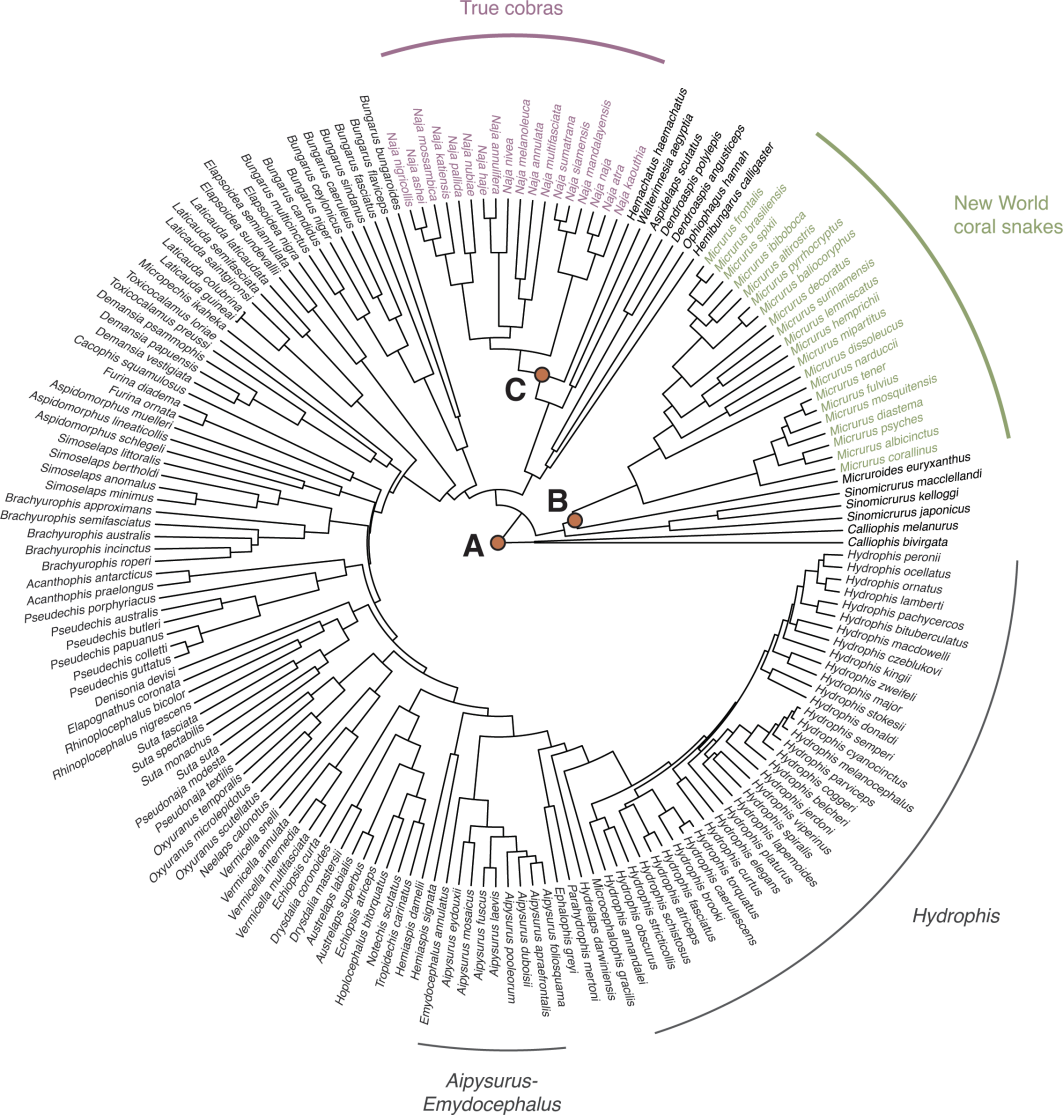
Elapidae phylogeny from Lee *et al.* [16] showing locations of significant shift in diversification rate derived from likelihood-based-ratio statistics implemented in SymmeTREE version 1.1. Marked nodes indicate a significant shift in diversification rate for the more speciose clade: (A) all elapids except for the Asian coral snakes (genus *Calliophis*) (*p*Δ_1_ = 0.028); (B) New World coral snakes (genus *Micrurus*) (*p*Δ_1_ = 0.038); (C) true cobras (genus *Naja*) (*p*Δ_1_ = 0.046).

Our topological tests for diversification rate variation across all sources of phylogenetic inference showed that the difference in the number of species between the two clades is not significant under the ERM model and when assuming the estimated rates of speciation and extinction for the Elapidae phylogeny (table 1). Whole-tree tests detected significant diversification rate variation (*M*_Σ_, *p* = < 0.001) across the elapid phylogeny, but not on the branch leading to the *Hydrophis* clade (Δ_1_ = 1.94, *p*Δ_1_ = 0.073). Significant shifts in diversification rate were detected on the lineages giving rise to: (i) all elapids except for the Asian coral snakes (genus *Calliophis*) (node A, figure 2; Δ_1_ = 2.84, *p*Δ_1_ = 0.028), (ii) New World coral snakes (genus *Micrurus*) (node B, figure 2; Δ_1_ = 2.87, *p*Δ_1_ = 0.038), and (iii) true cobras (genus *Naja*) (node C, figure 2; Δ_1_ = 2.71, *p*Δ_1_ = 0.046). These results contrast with the findings of Lee *et al.* [16] particularly concerning the *Hydrophis* clade.

### 3.2. Testing the greater degree of morphological disparity in *Hydrophis*

**Figure 3.**
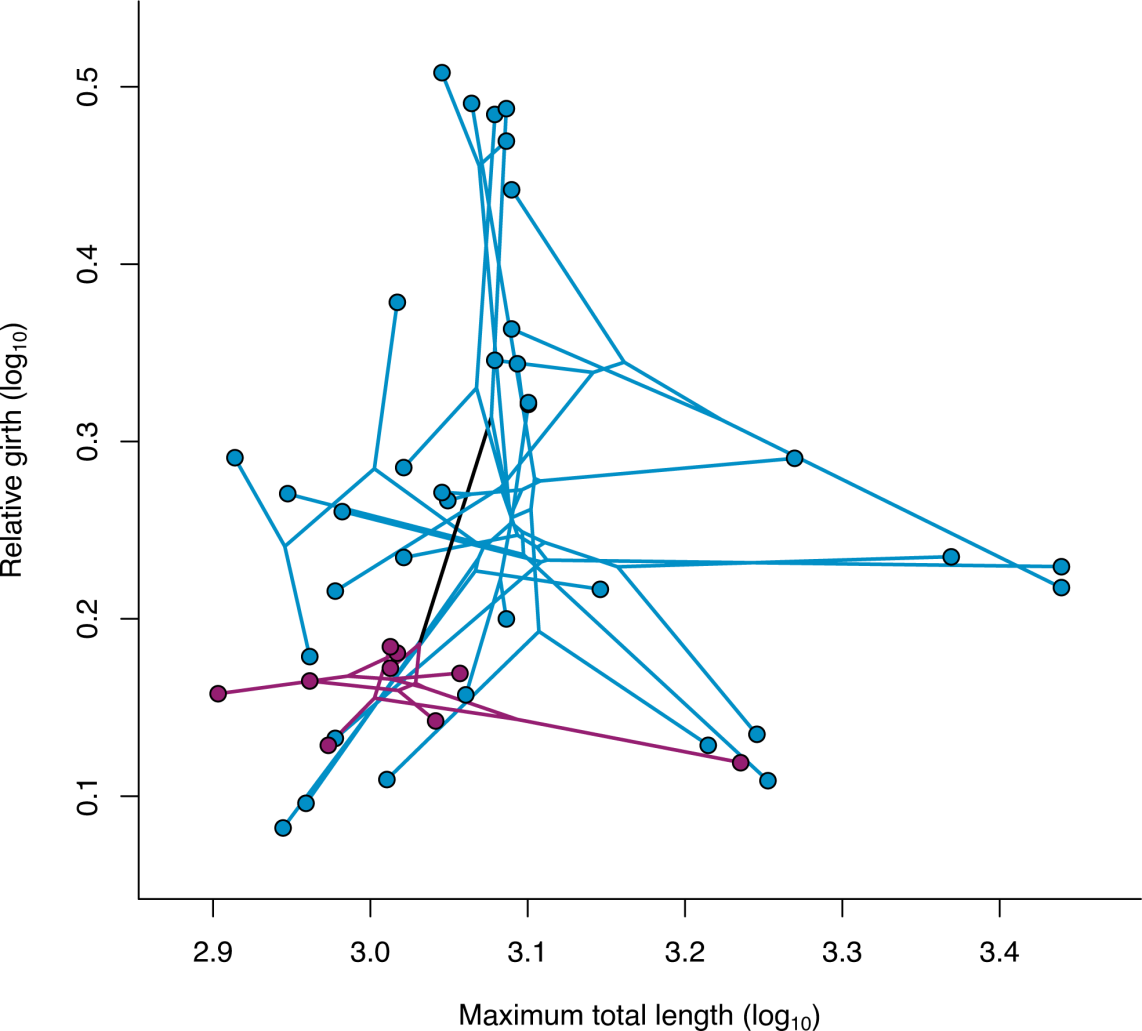
Phylomorphospace plot using phylogenetic tree and morphological trait dataset from Sherratt *et al* [34], showing region occupied by *Hydrophis* species (blue) and *Aipysurus-Emydocephalus* species (purple).

Phylomorphospaces of *Aipysurus-Emydocephalus* and *Hydrophis* groups are visualized in figure 3. Direct comparison of the mean morphometric branch length values for the *Aipysurus-Emydocephalus* and the *Hydrophis* clades indicated that lineages within *Hydrophis* have a greater magnitude of morphological change than *Aipysurus-Emydocephalus* (*M*_Hyd_ = 169.55; *M*_AE_ = 108.05). However, the observed *M*-ratio (*M*_Hyd_/*M*_AE_ = 1.57) was found to be non-significant (*p* = 0.06) (table 2). Using raw measurements of morphological traits, a lower lineage density (i.e. greater dispersion across the morphospace) was observed in *Hydrophis* (*D*_1Hyd_ = 2.61) than *Aipysurus-Emydocephalus* (*D*_1AE_ = 9.80) and was supported with a significant *D*-ratio (*D*_1AE_/*D*_1Hyd_ = 3.75; *p* =<0.001). Similar lineage densities and a significant *D*-ratio were found with transformed morphological trait data (log_10_-transformed data: *D*_1AE_ = 38.53; *D*_1Hyd_ = 21.61; *D*_1AE_/*D*_1Hyd_ = 1.78, *p* = 0.005) (table 2). Histograms showing simulated *M*- and *D*-ratio values and where observed *M*- and *D*-ratios sit in the distribution are provided in the electronic supplementary material (electronic supplementary material, figures S1–S3).

**Table 2.** Tests of unequal magnitude (mean morphometric change per phylogenetic branch) and mode (lineage density) of morphological diversification between *Aipysurus-Emydocephalus* and *Hydrophis* clades. Asterisk indicates significance at *α* = 0.05.

clade	magnitude		mode			
	*M*	*M*_Hyd_/*M*_AE_	*D*_1_	*D*_1AE_/*D*_1Hyd_	*D*_1_ ^a^	*D*_1AE_/*D*_1Hyd_^a^
*Aipysurus-Emydocephalus*	108.05	1.57	9.80	3.75*	38.53	1.78*
*Hydrophis*	169.55		2.61		21.61	

^a^ Lineage density values and ratio calculated from log_10_-transformed data.

## 4. Discussion

In this study, we re-examined the basis of claims for a rapid adaptive radiation of the *Hydrophis* lineage: (i) an unusually high number of species and (ii) a greater degree of morphological diversity. By using appropriate null models, we showed that the *Hydrophis* group of sea snakes is not exceptionally species-rich and that there is no evidence to support a significantly greater diversification rate compared to the *Aipysurus-Emydocephalus* group. In contrast, the results of our phylomorphospace analyses confirm that the *Hydrophis* lineage occupies a significantly larger region of possible morphologies than the *Aipysurus-Emydocephalus* clade. We demonstrated that *Aipysurus-Emydocephalus* species have evolved a constrained suite of morphological trait values, while *Hydrophis* species reflect the occupation of novel regions of the morphospace throughout its diversification history consistent with greater morphological innovation. More importantly, we gained evidence to indicate that the *Hydrophis* lineage has likely undergone a non-Brownian Motion mode of morphological diversification, which is consistent with the claim of this group as a model of an adaptive radiation in the marine environment under the criterion of exceptional morphological diversification.

Our topological tests of diversification rate variation reveal that the *Hydrophis* clade does not have a substantially greater number of species relative to the *Aipysurus-Emydocephalus* lineage and that there is no evidence for differential diversification rates between the two clades. Indeed, observed topological patterns based on several sources of phylogenetic inference cannot be distinguished from an ERM model of diversification. Therefore, the observed difference in species numbers between *Aipysurus-Emydocephalus* and *Hydrophis* does not require a deterministic explanation and may be explained most parsimoniously as a product of the stochastic nature of diversification. This outcome contrasts findings of Sanders *et al.* [37] that suggested a significant difference in species richness between *Aipysurus-Emydocephalus* (9 species) and *Hydrophis* (48 species) based on a similar test, which includes a temporal component [38]. While such tests add a temporal aspect, and thus more assumptions into the evaluation, the lack of a clear fossil record for true sea snakes [13] as well as the prevalence of cryptic species in the *Hydrophis* clade can sway the results to favour a scenario where an excess of cladogenesis occurred in a short amount of time.

We also employed a whole-tree approach to test if significant shifts in diversification rate can be detected on the internal branch leading to the *Hydrophis* group. As with our topological tests, there was no evidence for a significant diversification rate shift specific to the *Hydrophis* lineage. This result conflicts with the findings of Lee *et al.* [16], who found shifts characterized by unusually strong accelerations in the rate of speciation on the internal branch leading to the *Hydrophis* clade. Inherent difference between the methods used in Lee *et al.* [16] (model selection using priors in BAMM; [39]) and this study (null model testing (ERM) in SymmeTREE) may have influenced such a discrepancy in results. However, extracting more information from trees, particularly estimating separate speciation and extinction rates, such as in BAMM, has been shown to provide misleading results [23]. Our approach, which uses conservative assumptions, avoids spurious interpretations regarding the diversification rate characteristics of the clades under study. Altogether, results of both our tests for diversification rate variation failed to satisfy the ‘rapid speciation’ criterion for adaptive radiations set by Schluter [2].

In our phylomorphospace analyses, we found no support that the *Hydrophis* clade evolved under a different tempo of morphological diversification compared to *Aipysurus-Emydocephalus*. Indeed, such temporal bursts in trait evolution were found to be rare in comparative data and may even be an unnecessary feature of adaptive radiation [40]. Other methods such as posterior predictive approaches may increase the power of detecting accelerated rates of morphological diversification especially when the rate of decline is weak [41].

Conversely, our analyses on lineage density provide evidence of a non-Brownian Motion mode of morphological diversification in *Hydrophis* snakes. Our results suggest that species of *Hydrophis* may have separated along the trait axes used here (body size and body shape) to make niches and use new resources. Our findings are consistent with existing conclusions on how the evolution of microcephaly uniquely among *Hydrophis* species has allowed for their access to prey with specific habits and for their use of novel resources in the marine environment [17]. The significantly reduced lineage density in the *Hydrophis* lineage relative to the *Aipysurus-Emydocephalus* group indicates its greater expansion into morphospace regions of possible and novel morphologies such as the evolution of smaller head sizes for specific species (i.e. microcephaly), and starkly contrasts with the *Aipysurus-Emydocephalus* group that has limited morphological disparity among its species despite occurring in ecologically similar marine environments. Collectively, such spread in the use of the available resource niche spaces and eventual divergence into multiple species consisting of a variety of specialized forms correspond to two other criteria of adaptive radiations: phenotype-environment correlation and trait utility [2].

Here we find that *Hydrophis* sea snakes satisfy three of the criteria set by Schluter [2] for identifying adaptive radiations: (i) common ancestry, (ii) phenotype-environment correlation, (iii) trait utility. However, they do not satisfy the criterion of diversification rate change (i.e. rapid speciation). Nonetheless, we propose that the *Hydrophis* clade remains a putative case of adaptive radiation in the marine environment because it represents the origination of species with accompanying adaptations. It is important to note that some proponents of adaptive radiation did not include rapid speciation (or explosive diversification) as a defining characteristic of adaptive radiations (reviewed in Givnish [6]). Another view suggests that macroevolutionary patterns lie somewhere in the radiation continuum [42]. Clades may be categorized as one of the following: adaptive radiation, non-adaptive non-radiation, non-adaptive radiation or adaptive non-radiation. However, such categories mainly emphasize the rates of diversification and phenotypic evolution; where the difference between adaptive and non-adaptive relies on how high or how low the rate of phenotypic evolution is, and where the difference between a radiation and a non-radiation depends on how high or how low the rate of diversification is. Our findings on the *Hydrophis* lineage may be akin to the definition of an ‘adaptive non-radiation’ (*sensu* Morinaga *et al.* [42]), but specific formal testing under such framework is required as the limits and the range of rates defining these categories vary across organisms [42].

We support the view that adaptive radiations are not necessarily rapid and that cases where rapid speciation is observed do not necessarily qualify as adaptive radiations. The attribute of rapid speciation was only cited by Simpson [3] but in the same work, Simpson [3] also pointed out that adaptive radiation may happen gradually. Moreover, rapid speciation may proceed through other factors such as limited dispersal or sexual selection, and such processes do not necessarily lead to the adaptive divergence of taxa from a single ancestral type [6]. Therefore, rapid speciation or explosive diversification including the number of species within a clade should not take precedence [43] or should not even be considered [1] as a criterion in defining and identifying adaptive radiations. As an example, Darwin’s finches do not exhibit significantly elevated net species diversification relative to closely related coerebid birds [44] and only comprise 15 species [45], yet this study system is the paradigmatic case of adaptive radiation. This viewpoint of adaptive radiation ultimately avoids its conflation with mere speciation and adheres to its central theme of diversification with adaptation.

Species formation without adaptation or vice versa is not considered adaptive radiation [1,6,7]. While there was no evidence for a burst in diversification, the formation of species in the *Hydrophis* clade produced biodiversity with corresponding ecological functions and/or roles that allowed its species to seize ecological opportunities. In this study, we accommodated the role of stochasticity in generating the observed disparate patterns of species richness and morphological diversity between the two major sea snake clades. We established that their difference in species numbers may be parsimoniously explained by random variation, while the dissimilarity in their degree of morphological diversity calls for a deterministic hypothesis. Explicitly testing the role of chance is fundamental in understanding the underpinnings of multicausal patterns in macroevolution such as adaptive radiations.

## Data Availability

Clade size information were obtained from the following: Squamate phylogenies [24,25]; sea snake phylogenies [12,14,16,34]; list of nominal species [26]. Morphological trait dataset was obtained from Sherratt *et al.* 2018 dataset [34]. Data and relevant code for this research work are stored in GitHub: https://github.com/grcvhon/sea-snake-macroevolution and have been archived within the Zenodo repository [46]. Supplementary material is available online [47].
